# Inequalities in sub-Saharan African women’s and girls’ health opportunities and outcomes: evidence from the Demographic and Health Surveys

**DOI:** 10.7189/jogh.09.010410

**Published:** 2019-06

**Authors:** Clara Pons-Duran, Anna Lucas, Ambar Narayan, Andrew Dabalen, Clara Menéndez

**Affiliations:** 1 ISGlobal, Hospital Clínic – Universitat de Barcelona, Barcelona, Spain; 2 Poverty and Equity Global Practice, The World Bank Group, Washington, D.C., USA

## Abstract

**Background:**

Maternal and reproductive health services are far from universalization and important gaps exist in their distribution across groups of women in sub-Saharan Africa (SSA). The aim of this study is to determine the magnitude of this unequal distribution of maternal and reproductive health-related opportunities and outcomes and to identify the major sources of inequality.

**Methods:**

Demographic and Health Surveys data were used to analyse 15 opportunities for women of reproductive age (15-49), pregnant women and older adolescent girls (15-19), across 29 SSA countries. The tool employed is the Human Opportunity Index (HOI), a composite indicator that combines the availability of an opportunity (the coverage rate) with a measure of how equitably it is distributed among groups of women with different characteristics (or circumstances). Decompositions are used to assess the contribution of each individual circumstance to inequality.

**Results:**

The maternity care package of services is found to have lowest average HOI (26%), while exclusive breastfeeding among children aged 0-6 months has the highest HOI (77%). The other indicators show low HOIs, sometimes lower than 50%, indicating low coverage and/or high inequality. Wealth, education and area of residence are the main contributors to inequality for women of reproductive age. Among adolescent girls, marital status is the major contributor.

**Conclusions:**

Reproductive and maternal health opportunities for women in SSA are scarce and far from reaching the global goals set by the post 2015 agenda. Further progress in improving women’s and adolescents’ health and well-being can only be achieved by a strong expansion of coverage to produce a more equitable and efficient distribution of health care. Failure to do so will compromise the likelihood of achieving the post-2015 Sustainable Development Goals (SDG). New metrics such as the HOI allows better understanding of the nature of challenges to achieving equity in perinatal and reproductive health, and offers a tool for monitoring progress in implementing a strong equity agenda as a part of the SDG initiative.

Sub-Saharan Africa (SSA) is home to more than 230 million women of reproductive age, between 15 and 49 years [1]. In 2015, SSA accounted for roughly two-thirds of all maternal deaths in the world with a maternal mortality ratio (MMR) of 546 maternal deaths per 100 000 live births [2]. As in other regions in the world, universal health coverage (UHC) has not been achieved for most of the health services and interventions, including maternal health related ones [2]. Important disparities persist in access to maternal and reproductive health services among women in SSA across groups within and between countries [3-5]. Despite progress during the Millennium Development Goals (MDGs) period (2000-2015), it is estimated that half of women in SSA do not have access to the essential health care during pregnancy and childbirth and contraceptive use remains low and insufficient with only 28% prevalence in 2015 among women who are married or in union [3].

Scarcity by its very nature produces inequality between those who have access and those who do not, which is often manifested as systematic and persistent gaps between individuals belonging to different socioeconomic groups [6]. When services are insufficient, an individual’s chances of accessing them are likely to be influenced by her circumstances, the economic and social attributes of the individual and her family. This in turn produces inequalities in access to services between groups differentiated by these circumstances. These characteristics can be seen as the social determinants of health status [6,7].

Opportunities are understood as the minimum set of essential goods and services that enable individuals to realize their human potential, a definition that has been applied in existing World Bank studies on opportunities for children mainly, but also for other population groups [6-9]. The concept of equality of opportunity requires that individuals’ opportunities are independent of their life circumstances [10,11], which are the characteristics that an individual is born into, such as religion or wealth of one’s parents, and over which she has no influence. Opinions differ about what constitutes opportunities for adults, since unlike in the case of children, an adult’s access to basic services would depend at least in part on her own choices and decisions. However, there is a strong rationale for considering certain types of essential services or indicators of well-being as opportunities even for adults, particularly for women in SSA, whose choices are constrained by circumstances that are mostly outside their control.

Studies have shown that almost all maternal and reproductive health opportunities are unequally distributed among population groups with different wealth characteristics, areas of residence or educational levels [4,5,12]. The current study aims to go one step further: to consider all such characteristics simultaneously to see the magnitude and sources of inequality for different indicators of access to health opportunities and outcomes, and thus identify which circumstances are associated with the highest inequalities in SSA. This method has only been used once with maternal and reproductive health indicators in a peer-reviewed publication [13]. The consistent method applied to the analysis in multiple countries, using similar sources of data, also allows for aggregation across countries. This analysis covers all women of reproductive age, with an additional focus on older adolescent girls – those between 15 and 19 years old – and on pregnant women.

This paper is based on the report *“Inequalities in women’s and girls’ health opportunities and outcomes: A report from sub-Saharan Africa”* that was published in December 2016 jointly by the Barcelona Institute for Global Health (ISGlobal) and the Poverty and Equity Global Practice of the World Bank Group, by the same authors [14].

## METHODS

### Defining opportunities

The selected indicators include health outcomes and the use or knowledge of health services (**Table 1**). For the study of older adolescent girls, education has also been selected as an opportunity because it seems to be associated with adolescents’ reproductive health, early marriages and high-risk pregnancies [15,16]. Some of the indicators are not completely related to health intervention coverage (eg, not having anaemia or having the recommended BMI), while others could be influenced by individual decisions or even chance. However, all of them have been treated as health opportunities for women since they are considered key aspects of maternal and reproductive health.

**Table 1:** List of opportunities and the baseline population for whom they have been analysed

Opportunity	Description
Not having anaemia	Women without anaemia
	**Baseline population**: all women of reproductive age (15-49)
Having the recommended BMI (18.5-24.99)	Women with a BMI between 18.5 and 24.99
	**Baseline population**: all women of reproductive age (15-49)
Met need for family planning	Women currently using contraceptive methods
	**Baseline population**: women of reproductive age (20-49) or older adolescent girls (15-19) with a need for family planning
Knowledge of a place where to get an HIV test	Women who know where to get an HIV test
	**Baseline population**: all women of reproductive age (15-49)
Four antenatal care visits attended by skilled personnel*	Women who received at least four antenatal care visits and report being attended by skilled personnel (doctor, nurse, midwife or auxiliary midwife)
	**Baseline population**: all women with newborns in the five years preceding the interview date
Delivery attended by a skilled attendant	Women who had a delivery attended by a doctor, nurse, midwife or auxiliary midwife
	**Baseline population**: all women with newborns in the five years preceding the interview date
Mother’s checkup after delivery	Women who had a checkup after delivery
	**Baseline population**: all women with newborns in the two/five years preceding the interview date
Maternity care package	Women who attended at least four antenatal care visits, had a delivery attended by skilled personnel AND had a checkup after delivery
	**Baseline population**: all women with newborns in the five years preceding the interview date
At least one dose of IPTp (SP)	Women who took at least one dose of IPTp (SP)
	**Baseline population**: all women with newborns in the five years prior to interview and received at least one antenatal care visit
HIV test offered during pregnancy	Women who were offered an HIV test during antenatal care visits
	**Base population**: all women with newborns in the two years prior to interview and received at least one antenatal care visit
Infant checkup within two months after delivery	Women whose last child had a checkup within two months after delivery
	**Base population**: all women with newborns in the two/five years prior to the interview date and the child survived
Exclusive breastfeeding among children 0-6 months	Women who are breastfeeding and are not giving the children any other type of food or beverage
	**Base population**: all women with newborns in the six months prior to the interview date and the child survived
Having never been pregnant	Women who have never had a child, a stillbirth or an abortion, or are not currently pregnant
	**Base population**: Older adolescent girls (15-19)
Currently attending school	Women who are currently attending school (or university)
	**Base population**: Older adolescent girls (15-19)

BMI – Body Mass Index, HIV – Human Immunodeficiency Virus, IPTp – Intermittent Preventive Treatment in Pregnancy, SP – Sulfadoxine-Pyrimethamine

*Information regarding attendance by skilled health personnel is recorded only once during the interview, therefore it is not specific of each ANC visit attended. This variable is, in fact, a proxy of the optimal indicator where information regarding each particular visit would be recorded. In addition, the definition of skilled health personnel has been homogenized to be able to compare the variables across countries. Only doctors, nurses, midwifes and auxiliary midwifes have been considered skilled.

Recognizing that having access to just one service is not enough to meet the standards of basic opportunities in maternity care, a “composite” indicator for maternity care is also constructed, where the opportunity is defined as having access to all three basic services for a pregnant woman: attending at least four antenatal care visits, having a delivery attended by skilled personnel, and having a check-up after delivery.

### Defining circumstances

The list of selected circumstances can be categorized into five groups (**Table 2**). The majority of the circumstances are used in the analysis of all women of reproductive age, but age is substituted by age at delivery for the analysis of pregnancy related opportunities. For the analysis of older adolescents’ opportunities, the list varies as shown in **Table 2**. The categorization of all circumstances can be found in **Table 3**.

**Table 2:** List of circumstances*

	Women of reproductive age	Pregnant women	Older adolescent girls
**Women’s characteristics**	Age	Age at delivery	-
	Marital status	Marital status	Marital status
	Number of children	Number of children	-
**Household head characteristics**	Sex of the household head	Sex of the household head	Sex of the household head
**Socio-cultural background**	Religion	Religion	Religion
	Educational level	Educational level	-
	-	-	Occupational status
**Location**	Area (urban/rural)	Area (urban/rural)	Area (urban/rural)
**Household status**	Wealth index† (quintiles)	Wealth index† (quintiles)	Wealth index† (quintiles)

*The set of circumstances for Niger and Tanzania does not include religion, and the one for Mali and Senegal does not include occupational status, because these data were not available.

†It is obtained as an index, computed by the Demographic and Health Surveys program, from assets held by households and living conditions that are associated with wealth.

**Table 3:** Categorization of the circumstances

Circumstance	Categories
Age	*Continuous variable*
Age at delivery	*Continuous variable*
Area	Urban
	Rural
Educational level	No schooling
	Primary school
	Secondary school
	Higher education
Marital status	Never married or in union
	Currently or previously married or in union
Number of children	*Continuous variable*
Occupational status	Not working
	Working
Religion	Non-religious
	Muslim
	Christian
	Animist /traditional religion
	Others/unclassified
Sex of the household head	Male
	Female
Wealth index*	1st quintile (the poorest)
	2nd quintile
	3rd quintile
	4th quintile
	5th quintile (the wealthiest)

*Computed by the Demographic and Health Surveys program, the wealth index is obtained from assets held by households and living conditions that are associated with wealth.

Although the drivers of inequality can be very different across countries, it has been considered that across SSA they are similar and therefore, a single set of circumstances has been selected in each case for the whole group of study countries. As it explained below, this homogenization is needed for comparability purposes (see The Human Opportunity Index section).

### Study design, data sources and study population

This study analyses the inequality of opportunity of 15 reproductive and maternal health indicators in 29 SSA countries (**Table 4**). See Table S1 in **Online Supplementary Document** for more information, including as sample sizes. The data sources for this study are the Demographic and Health Surveys (DHS) financed by the United States Agency for International Development (USAID) [17]. The countries included in the analysis are those having at least one available standard and complete DHS conducted between 2010 and 2015. The most recent data set of each country available when the research was undertaken (March 2016) were selected for the study (**Table 4**).

**Table 4:** List of countries and DHS surveys*

	Country	Survey year	African UN region		Country	Survey year	African UN region
1	**Benin**	2011-2012	Western	16	**Malawi**	2010	Eastern
2	**Burkina Faso**	2010	Western	17	**Mali**	2012-2013	Western
3	**Burundi**	2010	Eastern	18	**Mozambique**	2011	Eastern
4	**Cameroon**	2011	Central	19	**Namibia**	2013	Southern
5	**Comoros**	2012	Eastern	20	**Niger**	2012	Western
6	**Congo Rep.**	2011-2012	Central	21	**Nigeria**	2013	Western
7	**Congo DR**	2013-2014	Central	22	**Rwanda**	2014-2015	Eastern
8	**Cote d'Ivoire**	2011-2012	Western	23	**Senegal**	2014	Western
9	**Ethiopia**	2011	Eastern	24	**Sierra Leone**	2013	Western
10	**Gabon**	2012	Central	25	**Tanzania**	2010	Eastern
11	**The Gambia**	2013	Western	26	**Togo**	2013-2014	Western
12	**Ghana**	2014	Western	27	**Uganda**	2011	Eastern
13	**Guinea**	2012	Western	28	**Zambia**	2013-2014	Eastern
14	**Kenya**	2014	Eastern	29	**Zimbabwe**	2010-2011	Eastern
15	**Liberia**	2013	Western				

DHS – Demographic and Health Surveys, Congo DR – Democratic Republic of Congo, Congo Rep. – Congo Republic, UN – United Nations

*The research was undertaken in March 2016.

The study population is comprised of women of reproductive age between 15 and 49 years of age. Different subgroups of this population, pregnant women and older adolescent girls between 15 and 19 years old, are then used to analyse certain indicators that are only relevant for that specific subgroup (**Table 1**).

### The Human Opportunity Index

**Figure 1:**
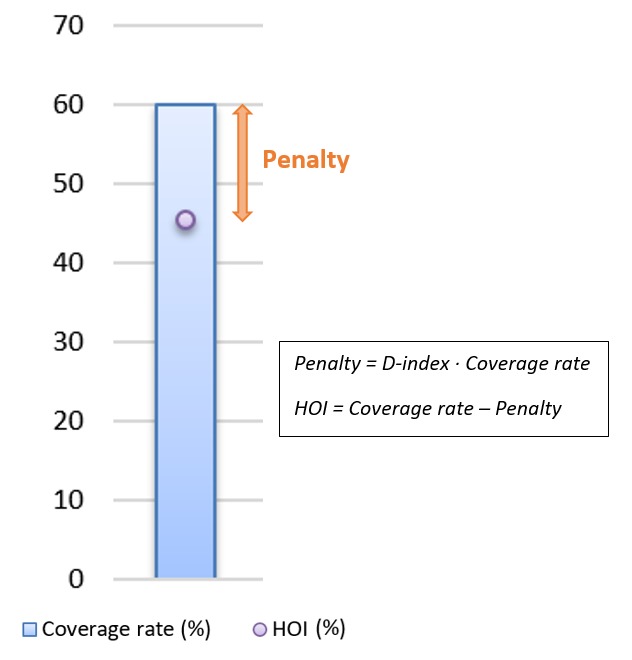
How to interpret the HOI. Note: HOI – Human Opportunity Index, D-index – Dissimilarity index.

The Human Opportunity Index (HOI) is a measure of the coverage rate of an opportunity – ie, number of individuals that have an opportunity over all individuals in need –, discounted by inequality in its distribution across circumstance groups – ie, sets of individuals with the same circumstances. It summarises two elements in a synthetic indicator: how many opportunities are available (the coverage rate), and how equitably those opportunities are distributed across groups defined by their circumstances. If the coverage rate is close to the HOI, the distribution of the opportunities is equitable; when the HOI is lower than the coverage rate, the gap between them indicates inequality (**Figure 1**). The HOI was developed by the World Bank with external researchers and first presented in 2009 [7,8].

The HOI (*H*) for a particular opportunity is the coverage rate for this opportunity (*C*) discounted by a penalty (*P*) due to inequality in coverage between population groups with different circumstances:

H = C – P (1)

Alternatively, the HOI can be expressed as the coverage rate multiplied by a factor of equality:


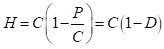
(2)

Where 1 – *D* is equal to one if access to the opportunity is independent of the circumstances, in which case the HOI is equal to the average coverage rate. *H* can be interpreted as the share of the total number of opportunities that needs to be reallocated between circumstance groups to ensure equality of opportunities, which we refer to as the dissimilarity index (D-Index) or the inequality of opportunity index [7,18]. For each circumstance group, *D* can be computed as follows:


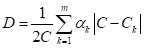
(3)

where *k* is a group with a specific set of circumstances, *C_k_*the coverage rate of group* k, α_k_*the share of group k in total population; and *m* the numbers of groups defined by circumstances (each group consists of all individuals who share the same circumstances). The D-index would be 0 in the hypothetical situation of perfect equality of opportunity. The maximum value it can take is 1 (or 100 when talking about percentages).

When analysing household survey data with multiple circumstances, and categories within, the formula in (3) cannot be applied directly because of limited number of observations within each circumstance group, with some of the circumstance groups even being a null set in some cases. Instead, an econometric procedure is used to obtain an estimate of *D* (and thus *H*) [9,13]. Coverage rates in the formula (3) are substituted by probabilities. This procedure consists of running a logistic regression model to estimate the relationship between access to an opportunity (dependent variable) and the circumstances of an individual (independent regressors), on the full sample of individuals. All independent factors (circumstances) are included at the same time in the regression. The estimated coefficients of the regression are used to obtain the predicted probability of access for each individual in the sample. These predicted probabilities (*p*) together with the sample size (*n*) are used through the following equation to calculate the predicted overall coverage (Ĉ) of a specific opportunity:


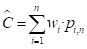
;(4)

where 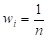.

With *p* and *Ĉ* we can compute the predicted D-index (D):


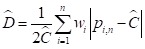
(5)

More details on the estimation and properties of the HOI and the D-index are available elsewhere [6,7,13].

### Shapley decomposition

The D-index is a function of the set of circumstances used to define groups and can therefore change as the set of circumstances changes. In particular, *D *cannot decrease in value when more circumstances are added to any existing set of circumstances. This in turn implies that the measured D-Index is always a lower bound of the actual inequality that would be estimated if one could observe and use all relevant circumstance variables. This property also allows defining the contribution of each circumstance to inequality as the increase in D-index due to the addition of a circumstance, or the marginal value added by a new circumstance, to the D-Index [6].

Given the property that D-Index increases with the number of circumstances, the Shapley decomposition of the index, first described by Shorrocks in 2012 [19] according to the Shapley value concept, can be conducted to estimate the relative contribution of each circumstance to the inequality index. In these decompositions, contributions add up to the value of the D-index computed with all the available circumstances in the data (all the regressors included in the above-mentioned logistic regression) [6]. The contribution of every circumstance is estimated as the weighted average of all (proportional) changes in the index that are induced by adding each circumstance to all possible permutations for all possible subsets of the other circumstances. Circumstances that add higher marginal value to the D-Index are interpreted as contributing a larger share to the inequality between groups [6]. Detailed information on the construction, properties and limitations of the Shapley decomposition and Shapley value is available elsewhere [7,20].

### Data management and statistical software

The country-level analyses were weighted using the sample weights available in the DHS programme data sets [17]. To obtain SSA average for an opportunity, simple unweighted mean from individual country results was computed. In addition, as a robustness check, pooled multi-country analyses were performed weighting the countries according to the women of reproductive age population in each one. Country specific results and those from the pooled weighted analyses are not reported in this article; they can be found elsewhere [14].

Stata 14 (Stata Corp., College Station, Texas, USA) was the statistical software used to perform the analyses.

### Ethical considerations

The research was undertaken under the Helsinki declaration. The DHS data sets were obtained after asking permission to the organization. The collection of the data was done by the DHS program team under the approval of the pertinent committees [21].

## RESULTS

The final study sample is comprised of 381 057 women of reproductive age, although the population included in the analyses of each opportunity varies depending on the baseline population and the missing values. The missing values for each opportunity represent less than 5% of the observations of almost all country samples, except for Comoros and Sierra Leone in antenatal care visits and Zimbabwe in not having anaemia, that represent less than 15% of the observations, and Namibia in antenatal care visits that represent less than 25%.

**Figure 2:**
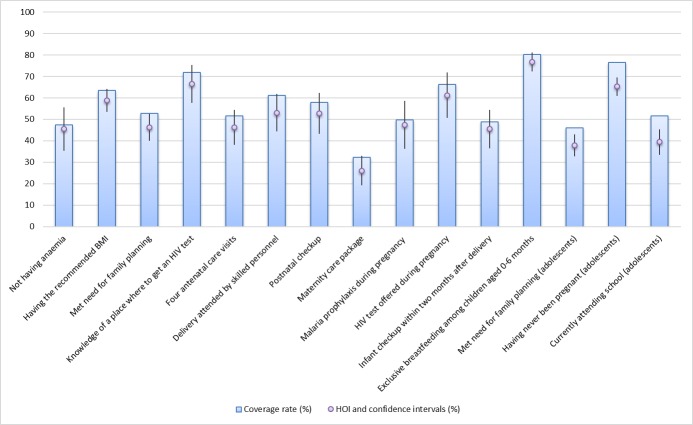
Average HOIs and coverage rates. Note: HOI – Human Opportunity Index, BMI – body mass index, HIV – human immunodeficiency virus.

The average HOIs and coverage rates (across countries, unweighted by population) of the opportunities included in the study are shown in **Figure 2** and the numerical values can be found in Table S2 in **Online Supplementary Document**. The distance between them represents the penalty for inequality of opportunity (which is *P* = C × D, using equations (1) and (2) (**Figure 1**). The lowest average HOI corresponds to the maternity care package (26.08, 95% confidence interval (CI) = 19.28-32.88), followed by the met need for family planning (37.82%, 95% CI = 32.66-42.98) and currently attending school among older adolescent girls (39.46%, 95% CI = 33.55-45.36). Exclusive breastfeeding among children aged 0-6 months has the highest average coverage rate (80.14%, 95% CI = 76.03-84.25) and average HOI (76.67%, 95% CI = 72.26-81.08). The lowest D-indices (inequality) correspond to not having anaemia (3.40%, 95% CI = 2.45-4.35) and exclusive breastfeeding (4.54%, 95% CI = 3.77-5.31). Except for the last two indicators, on average, the opportunities are low and unequally distributed across groups of women with different circumstances.

**Figure 3:**
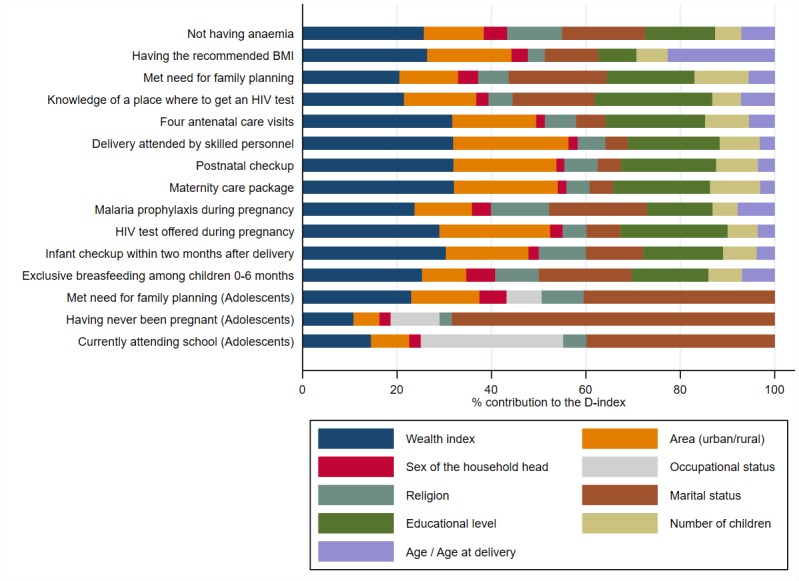
Average circumstances’ contributions to the D-index by opportunity. Note: D-index – Dissimilarity Index, BMI – body mass index, HIV – human immunodeficiency virus.

The Shapley decomposition results (**Figure 3**, unweighted averages across countries) show that family wealth status, education and area of residence are the factors that contribute the most to inequality for the majority of the opportunities for women of reproductive age. Age is an important contributor to inequality in having the recommended BMI (ranked just after family wealth status), which is an indicator for overall health status of a woman. The findings show that older women tend to display inadequate BMIs, generally high. Marital status stands as an important contributor to inequalities (D-index) for specific opportunities, with married women having the advantage over unmarried women for some indicators (eg, prophylaxis during pregnancy) and vice versa for others (eg, not having anaemia or met need for family planning).

**Figure 3** shows as well that among older adolescent girls, marital status is the major source of inequalities. The contribution of marital status is particularly high for the indicator of having never been pregnant, accounting for two thirds of the D-index. After marital status, wealth, area, and occupation are important contributors to inequality of opportunity among adolescent girls. For school attendance, occupation is the second most important source of inequality, which reflects that girls who work outside home are less likely to be in school. The D-index values for all the opportunities can be found in Table S2 in **Online Supplementary Document**.

**Figure 4:**
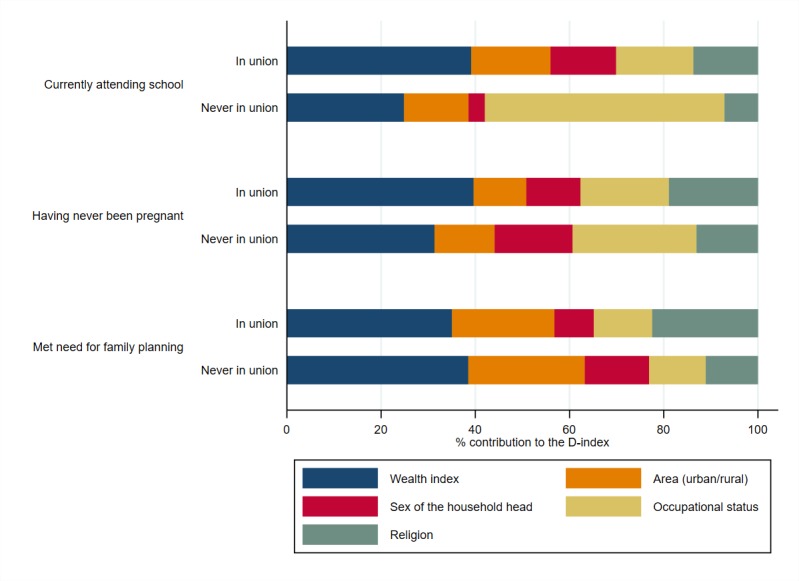
Older adolescents’ opportunities by marital status: average circumstances’ contributions to the D-index. Note: D-index – Dissimilarity Index.

The relatively large association of marital status and adolescents’ access to opportunities might have the effect of muting the contributions of the other circumstances on the D-index. To investigate further the role of these other circumstances, the sample is split into “in union” adolescents – married, divorced, living with a partner, or widowed – and “never in union”. Family wealth is the most important contributor to inequality in all opportunities for adolescents, except for the case of those currently attending school among “never in union” adolescents, where occupation is the main contributor to inequalities (or to the D-index) (**Figure 4**). The HOIs and D-indices of the split analysis by marital status can be found in Figure S1 in **Online Supplementary Document****.**

The weighted multi-country pooled analysis is consistent with the results shown in this article; coverage rates and HOIs are low while inequalities are high, and wealth, education and area of residence are the main contributors to inequalities [14]. The only fact that needs to be highlighted is the presence of religion as a relevant contributor to inequalities for some indicators (anaemia and malaria prophylaxis). This finding could suggest that religion is a confounding factor of the association between these indicators and geographical location.

## DISCUSSION

This study offers a novel approach to understand inequality of opportunities in reproductive, maternal, newborn, child and adolescent health by simultaneously analysing all possible factors for which data are available (which includes wealth, education, place of residence, religion, marital status and age), that matter for inequality in access to services among different groups, and the relative contribution of each factor to these inequalities.

This study highlights the challenges that women in SSA still face when seeking reproductive and maternal health services. On average, the HOIs obtained in the analysis appear to be low while inequalities are high. Notably, coverage is lower, and inequalities higher, for those interventions that require higher provider-patient interaction (eg, antenatal care or delivery attended by skilled personnel) than for interventions that can be delivered through strategies outside the health system (eg, exclusive breastfeeding or HIV information and testing), thus highlighting the importance of strengthening health systems to ensure equitable access. The indicator for (not having) anaemia is notable for showing low coverage *and* low inequality, whereas low coverage in other cases is almost always accompanied by high inequality. It is important to remark that differences in anaemia incidence, as it happens for all health outcomes, could be due also to other factors than inequality. Thus incidence of anaemia among women in SSA is high on the average and tends to affect all socio-economic groups within countries at nearly similar rates. This might suggest that anaemia has less to do with differential access to health services than other systemic country or region level factors (including exposure to specific diseases), identifying which will require more in-depth research. In contrast, inequality is higher for the opportunity of having the recommended BMI, which suggests large differences in general health between different groups of women in SSA countries, where family wealth status and age of the woman seem to matter the most for these difference.

The findings suggest that wealth, educational level and area of residence (urban/rural) are the three main circumstances associated with inequality of access to health care by women. Importantly, marital status appears as the main contributor to inequality among older adolescent girls, and only once marital status is controlled for, wealth becomes most important factor in the generation of inequality, similar to what is observed for adult women. Notably, all of these socio-economic barriers are often interlinked – women in poor families are also likely to be less educated and more likely to be living in rural areas. But the fact that education and location contribute a lot to inequality even after the role of economic status (family wealth) is accounted for suggests that these circumstances are important in their own right, net of the effect of economic status.

In order to interpret all these results, a couple of important considerations should be taken into account. The relationships above presented should not be interpreted as causal, but rather as providing information about associations between individual and household circumstances and inequality; more context-specific research tracing causal relationships will thus be needed to identify the determinants of inequalities in order to design the most appropriate interventions to address the observed inequities. Regarding the methodology used in the analysis, the HOIs and the D-indices are always upper and lower boundaries respectively, as they depend on the set of circumstances included in the analysis. The results for D-index are thus best interpreted as the most conservative estimates of inequality between groups – and the associated HOIs as the most liberal estimates of inequality-discounted coverage – which will only increase if other important yet unobserved circumstances could be included [6,7].

The main strengths of the study are listed as follows: the large number of countries and observations included in the analysis provides strong statistical power to the study; the DHS samples used are representative of the populations they include; and the average results of the 29 countries included in the study allow for drawing policy implications that can be generalised for SSA.

The main limitation of the study is the fact that the opportunities analysed here are likely to be affected by factors we cannot control for such as individual efforts and decisions or biological factors in the case of health outcomes, and therefore may not represent “opportunities” in the strict economic sense. Specifically regarding individual decisions, we have argued that inequalities between groups with different circumstances are still important to quantify, since the circumstances that drive these inequalities also effectively constrain the choices available to a woman in SSA. The inequalities we observe, therefore, can be interpreted as reflecting the association of circumstances with both unequal (physical) access and constrained (behavioural) choices. While our analysis cannot distinguish between these two channels, examining their relative roles in linking circumstances to outcomes should be an important topic for future research, so that inequities in health service delivery can be addressed by policies that are appropriately designed to address the underlying causes.

Another limitation of the methodology used is the fact that it does not give information about the direction of inequality. The HOI and the D-index highlight the presence of inequalities, but their direction has to be analysed in more detail in further analyses. However, this fact does not undervalue this research since the quantification of inequalities by itself sheds light on the maternal and reproductive health aspects that deserve more attention.

Finally, by using the same set of circumstances for the analyses in all countries, the specific country estimates could not be as optimal and adjusted as desired since drivers of inequality are very site specific. However, to be able to perform a multi-country analysis, and to compute comparable HOIs for policymaking in the region, it was necessary to select a single set of circumstances. The authors considered that it reflects well the majority of the country specific circumstances that drive inequality across SSA.

## CONCLUSIONS

Despite notable progress in the last decade, reproductive and maternal health opportunities for women and girls in SSA are scarce, with half of women and girls not receiving essential services. Accelerated progress towards the improvement of women’s and adolescents’ health and well-being can only be achieved by expanding coverage and reducing inequalities. In the current context of progressive universalization of basic health care in many contexts, the application of an equity principle that prioritizes the expansion of services among underserved or excluded populations can have important implications for policy choices.

Ensuring progressive and equitable expansion of health coverage should be the cornerstone of efforts as it is key to achieving the SDG3 health-related targets – including the ones addressing reproductive, maternal, newborn and child health, communicable diseases and UHC – and beyond, particularly SDG4 (Inclusive and equitable education), SDG5 (Gender equality) and SDG10 (Reduced inequalities). Research has a key role to play to further ascertain the levels and causes of inequalities, bridge the existing data gaps for specific subgroups of vulnerable women and girls, as well as for monitoring progress and accountability towards realizing the 2030 goals.

## Additional Material

Online Supplementary Document
