# Supplementary material for: Inequalities in sub-Saharan African women’s and girls’ health opportunities and outcomes: evidence from the Demographic and Health Surveys
Source: J Glob Health. 2018 Nov 29;9(1):010410. doi: 10.7189/jogh.09.010410 (PMC6326483; doi:10.7189/jogh.09.010410)
Supplement: Online Supplementary Document [file jogh-09-010410-s001.pdf]

Table S1. Country data

Country	UN Region	Survey year[1]	Language[1] (A/F)	Economy [2]	IMR[2]	MMR[2]	Population of women 15-49[2]	HIV prevalence [2]	IPTp (2 or 3 doses) policy since[3]	Indicators not available in the DHS survey of the particular country and year[1]
Benin	Western	2011-2012	F	LIC	70	436	2,311,289	1.2	2005	-
Burkina Faso	Western	2010	F	LIC	70	417	3,579,459	1.1	2005	-
Burundi	Eastern	2010	F	LIC	64	808	2,187,036	1.6	-	-
Cameroon	Middle	2011	F	LMIC	64	652	4,978,935	5	2004	-
Comoros	Eastern	2012	F	LIC	60	365	178,949	-	2003	Not having anaemia
Congo	Middle	2011-2012	F	LMIC	40	494	979,629	3.1	2006	-
Congo DR	Middle	2013-2014	F	LIC	78	746	16,167,171	1.1	2004	-
Cote d'Ivoire	Western	2011-2012	F	LMIC	75	715	4,701,945	3.8	2005	-
Ethiopia	Eastern	2011	-	LIC	48	482	20,811,496	1.3	-	Malaria prophylaxis during pregnancy
Gabon	Middle	2012	F	UMIC	40	314	387,504	4.3	2003	-
The Gambia	Western	2013	A	LIC	49	730	439,525	1.9	2003	-
Ghana	Western	2014	A	LMIC	44	322	6,803,551	1.5	2003	-
Guinea	Western	2012	F	LIC	67	695	2,678,217	1.6	2005	-
Kenya	Eastern	2014	A	LMIC	37	525	10,853,576	5.3	1999	Not having anaemia
Liberia	Western	2013	A	LIC	57	762	1,002,431	1.2	2004	Not having anaemia
Malawi	Eastern	2010	A	LIC	58	629	3,297,665	11.7	1993	Postnatal checkup
Mali	Western	2012-2013	F	LIC	79	617	3,517,972	1.4	2003	Newborn checkup within two months after delivery, occupation
Mozambique	Eastern	2011	-	LIC	68	596	5,735,866	11	2006	-
Namibia	Southern	2013	A	UMIC	34	283	624,523	16.2	2005	-
Niger	Western	2012	F	LIC	62	619	3,636,832	0.6	2005	Religion
Nigeria	Western	2013	A	LMIC	74	821	39,172,542	3.3	2004	Not having anaemia

Rwanda	Eastern	2014-2015	F	LIC	33	304	2,905,877	2.8	2005 – until 2008	Malaria prophylaxis during pregnancy
Senegal	Western	2014	F	LMIC	42	323	3,546,400	0.5	2004	Not having anaemia, having the recommended BMI, HIV test offered during pregnancy, occupation
Sierra Leone	Western	2013	A	LIC	94	1460	1,492,597	1.5	2004	-
Tanzania	Eastern	2010	A	LIC	42	514	10,532,046	6.1	2001	Postnatal checkup, religion
Togo	Western	2013-2014	F	LIC	55	386	1,689,457	2.5	2003	-
Uganda	Eastern	2011	A	LIC	46	408	7,460,696	7.1	2000	-
Zambia	Eastern	2013-2014	A	LMIC	47	237	3,476,200	12.6	2001	Not having anaemia
Zimbabwe	Eastern	2010-2011	A	LIC	56	446	3,551,962	18	2004	-

Note*: all country data belong to the year of the particular survey. UN=United Nations, A/F=Anglophone/Francophone, IMR=Infant Mortality Rate, MMR=Maternal Mortality Ratio, HIV=Human Immunodeficiency Virus, IPTp=Intermittent Preventive Treatment of malaria in pregnancy, DHS=Demographic Health Surveys, Congo=Congo Republic, Congo DR= Democratic Republic of Congo.*

References

1. USAID. The DHS Program. Available: http://www.dhsprogram.com. Accessed: 28 March 2018.

2. The World Bank Group. World Development Indicators. Washington DC: World Bank Group, 2018. Available: http://data.worldbank.org/data-catalog/world development-indicators. Accessed: 28 March 2018.

3. Van Eijk. A. M. et al. Coverage of malaria protection in pregnant women in sub- Saharan Africa: A synthesis and analysis of national survey data. Lancet Infect. Dis. 2011;11:190–207.

Table S2. Average HOIs, D-indexes and coverage rates by opportunity

Opportunities	Average HOI (CI)	Average D-index (CI)	Average Coverage rate (CI)
Not having anaemia	45,47 (35,32-55,63)	3,4 (2,45-4,35)	47,36 (36,91-57,80)
Having the recommended BMI	58,76 (53,44-64,07)	7,4 (6,10-8,70)	63,43 (58,08-68,79)
Met need for family planning	46,26 (39,89-52,64)	14,09 (11,40-16,79)	52,82 (46,94-58,70)
Knowledge of a place where to get an HIV test	66,48 (57,61-75,35)	9,36 (6,61-12,11)	71,84 (63,79-79,90)
Four antenatal care visits attended by skilled personnel	46,2 (38,07-54,32)	12,93 (9,48-16,38)	51,57 (43,62-59,53)
Delivery attended by skilled personnel	53,1 (44,43-61,76)	16,85 (12,20-21,51)	61,18 (53,11-69,26)
Mother's checkup after delivery	52,77 (43,24-62,30)	10,27 (6,90-13,65)	57,85 (48,21-67,49)
Maternity care package	26,08 (19,28-32,88)	24,64 (19,32-29,96)	32,4 (25,11-39,69)
*Malaria prophylaxis during pregnancy (*At least one dose of IPTp (SP))	47,45 (36,27-58,63)	7,47 (4,04-10,91)	49,74 (38,47-61,00)
HIV test offered during pregnancy	61,26 (50,74-71,79)	9,79 (6,27-13,32)	66,19 (56,21-76,17)
Infant checkup within two months after delivery	45,51 (36,52-54,50)	8,58 (5,98-11,18)	48,78 (39,66-57,91)
Exclusive breastfeeding among children aged 0-6 months	76,67 (72,26-81,08)	4,54 (3,77-5,31)	80,14 (76,03-84,25)
Met need for family planning (older adolescent girls)	37,82 (32,66-42,98)	19,23 (16,36-22,10)	45,99 (40,93-51,05)
Having never been pregnant (older adolescent girls)	65,27 (60,88-69,65)	15,18 (12,94-17,41)	76,43 (73,08-79,78)
Currently attending school (older adolescent girls)	39,46 (33,55-45,36)	26,38 (22,06-30,70)	51,57 (45,90-57,24)

Note: *D-index=Dissimilarity Index, BMI=Body Mass Index, HIV= Human Immunodeficiency Virus, HOI=Human Opportunity Index*

Figure S1. Older adolescents' opportunities by marital status: HOI and D-index by opportunity.

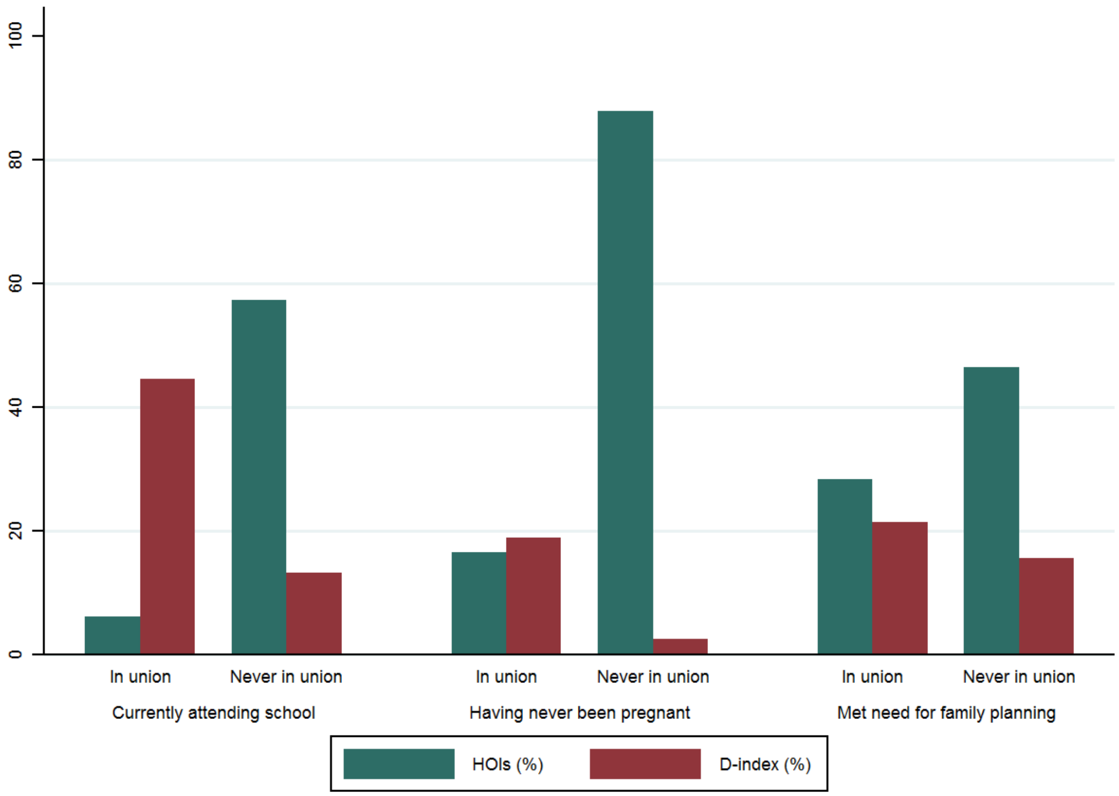

Note: *HOI=Human Opportunity Index, D-index=Dissimilarity Index*
